# Supplementary material for: Macroscopic weavable fibers of carbon nanotubes with giant thermoelectric power factor
Source: Nat Commun. 2021 Aug 13;12:4931. doi: 10.1038/s41467-021-25208-z (PMC8363648; doi:10.1038/s41467-021-25208-z)
Supplement: Supplementary file 1 — Supplementary Information [file 41467_2021_25208_MOESM1_ESM.pdf]

Supplementary Information for

Macroscopic Weavable Fibers of Carbon Nanotubes with Giant Thermoelectric Power Factor

Natsumi Komatsu[1], *Yota Ichinose*[2], *Oliver S. Dewey*[3], *Lauren W. Taylor*[3], *Mitchell A. Trafford*[3], *Yohei Yomogida*[2], *Geoff Wehmeyer*[4,5], *Matteo Pasquali*[3,5,6,7], *Kazuhiro Yanagi*[2], *Junichiro Kono*[1,7,8,*]

[1] Department of Electrical and Computer Engineering, Rice University, Houston, Texas, USA

[2] Department of Physics, Tokyo Metropolitan University, Tokyo, Japan

[3] Department of Chemical and Biomolecular Engineering, Rice University, Houston, Texas, USA

[4] Department of Mechanical Engineering, Rice University, Houston, Texas, USA

[5] Carbon Hub, Rice University, Houston, Texas, USA

[6] Department of Chemistry, Rice University, Houston, Texas, USA

[7] Department of Materials Science and NanoEngineering, Rice University, Houston, Texas, USA

[8] Department of Physics and Astronomy, Rice University, Houston, Texas, USA

*Corresponding author. Email: kono@rice.edu

Supplementary Note 1 Estimation of the position of E_F by optical spectroscopy studies

Thin films of aligned CNT were produced by a facile blade coating technique [1]. The raw CNT material was dissolved in CSA at a concentration of 0.5 wt%. The solution was pipetted onto a glass microscope slide and pressed between another slide to fully coat both slides. The slides sandwiched with solution were put into a custom-built poly(tetrafluoroethylene) (PTFE) holder. A PTFE push stick was used to quickly shear (at a rate of $\sim 10^4$ s^{-1}) the two glass sides apart from each other. This shearing force induced a uniaxial alignment of the CNTs. The films were then coagulated into acetone to remove the CSA and stabilize the aligned films.

The absorption spectra of CNT films were measured by a home-built optical measurement setup, consisting of a tungsten-halogen lamp (SLS201L, Thorlabs), a Glan-Thompson polarizer, and two spectrometers (covering 550-1080 nm and 1080-1425 nm). The spectral range of 1425-3300 nm was measured by Fourier transform infrared spectrometer (Nicolet™ iS50 FTIR Spectrometer, Thermo Fisher Scientific). The beam size was ~1 mm. Supplementary Figure 7 shows absorbance spectrum of the film for both cases where the incident light polarization direction and CNT alignment directions were parallel and perpendicular to each other.

For solution samples (Supplementary Figure 6), the raw CNT material was dispersed in pure water using sodium deoxycholate (Sigma-Aldrich). UV-VIS spectrophotometer (UV-1800, Shimadzu) was used for the spectrum range of 300-1080 nm, and the home-built setup was used for 1080-1300 nm.

Optical spectroscopy provides information on the nanotube diameter distribution and the position of E_F in each CNT sample; the latter is particularly important since the S value strongly depends on E_F [2,3]. Supplementary Figure 6 shows an absorbance spectrum for an aqueous suspension of the raw CNT material. The chiralities of the inner-wall nanotubes were assigned based on information available in the literature [2,3]. The diameters expected for the inner-wall nanotubes from Supplementary Figure 6 agree with results of our TEM analysis and are summarized in the top graph of Supplementary Figure 7.

Supplementary Figure 7a compares expected absorbance peak positions based on the diameter distribution determined by the TEM analysis with absorbance spectra of the four films that underwent the same chemical treatments as the four fibers in the TE measurements. Diameter information allows us to determine absorbance peaks originating from excitonic optical transitions [2,3], as shown in the top figure of Supplementary Figure 7a. Absorption peaks originating from the outer semiconducting tubes (S_{11} and S_{22}) are highlighted in red, those from the inner semiconducting tubes are highlighted in blue, and those from both the outer and inner metallic tubes (M_{11}) are in green.

The annealed films (annealed at 350 ˚C and 500 ˚C, respectively) show a peak at around 0.57 eV due to the S_{11} transition of the outer CNTs. A band of absorption peaks observed from 0.8 to 1.2 eV are due to a combination of the S_{22} peaks of the outer CNTs and the S_{11} peaks of the inner CNTs. Peaks are red-shifted compared to SWCNTs in an aqueous sodium dodecyl sulfate suspension (the top figure) [2,3]. The intensity of the S_{11} absorption band of the outer CNTs is lower than that of the S_{22} absorption band of the outer CNTs, suggesting that E_F is

likely to be inside the valence band, rather than inside the bandgap. Absorbance spectra for the as-produced and ICl-doped films are drastically different from those for the annealed ones, with the S_{11} and S_{22} peaks of the outer CNTs being suppressed due to Pauli blocking. The S_{11} peaks of the inner CNTs are still visible (from 1.0 to 1.2 eV), presumably because it is harder to dope the inner tubes, which are protected by the outer walls. For the perpendicular case of as produced and ICl doped samples, a new peak appears around 0.8 eV, considered to be the inter subband plasmon peak [4].

Supplementary Figure 7b schematically shows the E_{F} positions inferred from the results in Supplementary Figure 7a. The calculated density of states (DOS) is shown for the outer and inner CNTs as a function of energy. See below for the calculation details. For the film annealed at 500 ˚C, E_{F} is expected to be in the vicinity of the first van Hove singularity (VHS) of the outer semiconducting tubes, as discussed above. The E_{F} of the film annealed at 350 ˚C is expected to be located at a slightly lower energy than the 500 ˚C case because an outer S_{11} peak is still observable, but the film annealed at 350 ˚C has higher σ than the film annealed at 500 ˚C. The E_{F} of the as-produced film is in the vicinity of the first VHS of the inner semiconducting CNTs, as S_{11} peaks of inner CNTs are still visible. The E_{F} of the film doped with ICl is expected to be at a slightly lower energy than the as-produced film, because an inner S_{11} peak is still observable but the ICl doped film has higher σ than the as-produced film.

Supplementary Note 2 Theoretical Calculations

From TEM analysis, the average outer wall diameter was 1.78±0.219 nm, and the average inner wall diameter was 0.92±0.12 nm. We chose four representative single-wall carbon nanotubes (SWCNTs) in Supplementary Table 3 to describe double-wall carbon nanotubes (DWCNTs) used in this study.

We first calculated density of states DOS, conductance G, Seebeck coefficient S, and power factor $PF' \equiv GS^2$ for each SWCNT. Within the framework of the Landauer formula, the conductance $G(\mu)$ and Seebeck coefficient $S(\mu)$ with the chemical potential μ are expressed as

$$G(\mu) = \frac{2q^2}{h} \int dE\, T(E) \left(-\frac{\partial f(E,\mu)}{\partial E} \right) \tag{1}$$

and

$$S(\mu) = \frac{1}{qT} \frac{\int dE\, T(E) \left(-\frac{\partial f(E,\mu)}{\partial E} \right) (E-\mu)}{\int dE\, T(E) \left(-\frac{\partial f(E,\mu)}{\partial E} \right)}, \tag{2}$$

where q is the charge of carriers, h is the Planck constant, $f(E, \mu)$ is the Fermi–Dirac distribution function, and $T(E)$ is the transmission function. As can be seen here, we can obtain $G(\mu)$ and $S(\mu)$, once $T(E)$ is calculated.

We used the tight-binding method to calculate the thermoelectric properties of isolated SWCNTs in Supplementary Table 3 with 1×1×9 Monkhorst-Pack K-grid. The Slater–Koster parameters used in this method were obtained by the density-functional tight-binding method utilizing the "hotbit" code [5]. Using these parameters, we performed the calculation of density of states (DOS) for bulk SWCNTs. In addition, $T(E)$ of SWCNTs was calculated by combing the nonequilibrium Green's function method with the tight-binding method. SWCNTs were divided into three regions: device region, a left electrode, and a right electrode. The length of the device region is five unit cells for (11,0), (22,0), (6,6), and (13,13) SWCNT. The above-mentioned calculation was implemented in the ATK-SE package (Ver. 2017.2) [6]. Calculated DOS, G_{ind}, S_{ind}, and $PF'_{\mathrm{ind}} \equiv G_{\mathrm{ind}} S_{\mathrm{ind}}{}^2$ for each SWCNT are shown in Supplementary Figure 8.

As discussed in previous studies [7,8], peaks of S_{ind} of semiconducting SWCNTs appear inside the bandgap near the charge neutrality point (CNP), where chemical potential is 0 eV (Supplementary Figure 8a and b), while that of metallic SWCNTs appear near the first van Hove singularity (VHS) (Supplementary Figure 8d). On the other hand, the peaks of PF'_{ind} of both semiconducting and metallic SWCNTs are located near the VHS. Note that M1 does not show a peak in Supplementary Figure 8c solely due to limited chemical potential range. Although there is a nearly one order difference in S_{ind} values between semiconducting and metallic SWCNTs, the maximum values of PF'_{ind} are on the same order, which is consistent with Ref [7].

Next, we use simple circuit models suggested in [8–10] to approximate our DWCNT fiber. As shown in Supplementary Figure 9, one SWCNT is considered to be a combination of a generator ($S_i \Delta T$) and a resistor ($R_i = 1/G_i$), where i denotes that this is the ith nanotube in the system. For simplicity, we assume that temperature difference applied per one nanotube ΔT and Δt are the same for all nanotubes. The ratio of semiconducting to metallic was assumed to be 2:1 since we did not conduct any chirality enrichment. By using the parallel circuit model shown in Supplementary Figure 9b, the total Seebeck coefficient and conductance of the entire system can be written as [8–10]:

$$S = \frac{\sum_i S_i G_i}{\sum_i G_i} \quad \text{and} \quad G = \sum_i G_i \tag{3}$$

Thus, based on the ratio of semiconducting to metallic nanotubes (2:1), Seebeck coefficient S_p and conductance G_p of our system can be expressed as

$$S_p = \frac{2S_{\mathrm{SC1}} G_{\mathrm{SC1}} + 2S_{\mathrm{SC2}} G_{\mathrm{SC2}} + S_{\mathrm{M1}} G_{\mathrm{M1}} + S_{\mathrm{M2}} G_{\mathrm{M2}}}{2G_{\mathrm{SC1}} + 2G_{\mathrm{SC2}} + G_{\mathrm{M1}} + G_{\mathrm{M2}}}, \tag{4}$$

and

$$G_p = \frac{2}{6} G_{\mathrm{SC1}} + \frac{2}{6} G_{\mathrm{SC2}} + \frac{1}{6} G_{\mathrm{M1}} + \frac{1}{6} G_{\mathrm{M2}}, \tag{5}$$

where S_{SC1} is Seebeck coefficient of SC1 nanotube, G_{SC1} is the electrical conductance of SC1 nanotube, and similarly for SC2, M1, and M2 nanotubes.

For a series circuit model case as shown in Supplementary Figure 9c, the total Seebeck coefficient and conductance of the entire system can be written as [8–10]

$$S = \sum_i S_i \ \text{ and } \ \frac{1}{G} = \sum_i \frac{1}{G_i}. \tag{6}$$

For the series case, with a 2:1 ratio of semiconducting to metallic CNTs, the Seebeck coefficient S_s and conductance G_s of our system can be expressed as

$$S_s = \frac{2}{6} S_{\mathrm{SC1}} + \frac{2}{6} S_{\mathrm{SC2}} + \frac{1}{6} S_{\mathrm{M1}} + \frac{1}{6} S_{\mathrm{M1}}, \tag{7}$$

and

$$G_s = \left(\frac{2/6}{G_{\mathrm{SC1}}} + \frac{2/6}{G_{\mathrm{SC2}}} + \frac{1/6}{G_{\mathrm{M1}}} + \frac{1/6}{G_{\mathrm{M2}}} \right)^{-1}. \tag{8}$$

Supplementary Figure 10 shows states G_p, S_p, $PF'_p \equiv G_p {S_p}^2$, and DOS calculated for a parallel circuit case using Supplementary Equations (4) and (5). The chemical potential range examined was determined based on the optical study discussed in Supplementary Figure 7. G_p has finite values even inside the bandgap, as shown in Supplementary Figure 10a, because the total conductance of the system is simply the sum of the conductance from each contribution in the parallel case (Supplementary Equation (5)). S_p is drastically reduced especially inside the bandgap of SC1 and SC2 (Supplementary Figure 10b and e) because Supplementary Equation (4) can be approximated as

$$S_p \approx \frac{S_{\mathrm{M1}} G_{\mathrm{M1}} + S_{\mathrm{M2}} G_{\mathrm{M2}}}{G_{\mathrm{M1}} + G_{\mathrm{M2}}} \tag{9}$$

within the bandgap due to $G_{SC1} \approx G_{SC2} \approx 0$. Thus, instead of having peaks near the charge neutral point, peaks appear near VHS of SC1 and SC2 as discussed in Ref [8]. PF'_p has peaks in the vicinity of VHS as shown in Supplementary Figure 10c but smaller in amplitude compared to individual cases, again consistent with Ref [8]. The smaller amplitude is due to having metallic tubes and having two different diameter distributions. When sweeping chemical potential from 0 eV into bands (either negative or positive), the Supplementary Figure 10c shows that the first peak around ±0.2 eV is given by SC2 and the second peak around ±0.4 eV is given by semiconducting tubes (SC1 and SC2). Supplementary Figure 10f and g show S_p and PF'_p as a function of G_p respectively, showing multiple peaks in both showsS_p and PF'_p (mainly two peaks within the region examined) with increasing G_p.

Supplementary Figure 11 shows G_s, S_s, $PF'_s \equiv G_s {S_s}^2$, and DOS calculated for a series circuit case using Supplementary Equations (7) and (8). Conductance G_s is greatly reduced in the bandgap range of SC2, because G_s can be approximated as

$$G_s = \left(\frac{2}{G_{\mathrm{SC1}}} + \frac{2}{G_{\mathrm{SC2}}} + \frac{1}{G_{\mathrm{M1}}} + \frac{1}{G_{\mathrm{M2}}}\right)^{-1} \approx 0 \tag{10}$$

when $G_{SC1} \approx G_{SC2} \approx 0$ (Supplementary Figure 11a). Unlike parallel case, the maximum value of S_s is located near CNP, and its amplitude is comparable to SC2 case (Supplementary Figure 11b) because S_s is simply the sum of the Seebeck coefficient from each contribution in series case (Supplementary Equation (7)). PF'_s is almost zero in the bandgap of SC2 because G_s is largely suppressed in the range, and its amplitude is similar to the parallel case (Supplementary Figure 11c). Supplementary Figure 11f and g show S_s and PF'_s as a function of G_s respectively, showing a single peak within the region examined with increasing G_s, drastically different from the parallel case where there were multiple peaks (Supplementary Figure 10f and g).

The series case does not reproduce our experimental results well. Figures 1c and d demonstrates that both Seebeck coefficient S and power factor PF decreases with increasing conductivity σ, but Supplementary Figure 11g shows PF'_s increases with the increase of the conductance G_s. Furthermore, our gating measurement (Supplementary Figure 5) suggests that there is more than one peak in S and PF as a function of σ, contradicting to Supplementary Figure 11f and g.

The parallel case reproduces our data better, as pointed out in Ref. [8,11], but it has some discrepancies too. First, PF'_p as a function of G_p shows that the second peak is higher than the first peak (Supplementary Figure 10g), but Figure 1d suggests that PF monotonically decreases with σ. Second, as shown in Figure 2f, S value sharply increase right after the charge neutrality point, which Supplementary Figure 10a, b and c fail to reproduce.

Thus, we considered combining the parallel model and the series model, which is reasonable because there are both parallel and series connections existing inside our fiber. We used the model suggested in Ref [12] for combining the Seebeck coefficient,

$$S_{\mathrm{tot}} = (1-\beta)S_{\mathrm{s}} + \beta S_{\mathrm{p}}, \tag{11}$$

where S_{tot} is the combined Seebeck coefficient, S_{s} is the Seebeck coefficient from the series model, S_{p} is the Seebeck coefficient from the parallel model, and β is the fraction of parallel component, which will be optimized to describe our system the best. This equation of combining the series and parallel model is analogy to Supplementary Equation (6), where nanotubes were connected in the series way. Thus, we used the series model to combine conductance as the following:

$$G_{\mathrm{tot}} = \left(\frac{1-\alpha}{G_{\mathrm{s}}} + \frac{\alpha}{G_{\mathrm{p}}}\right)^{-1} \tag{12}$$

where G_{tot} is the combined conductance, G_{s} is the conductance from the series model, G_{p} is the conductance from the parallel model, and α is the fraction of parallel component, which will be optimized to describe our system the best.

We first tuned α while fixing $\beta = 1$. Supplementary Figure 12a and c show G_{tot} and PF'_{tot} with an α of 0 to 0.9 varied by 0.1 increments. As shown in Supplementary Figure 12a, G_{s} is dominant even at $\alpha = 0.9$, resulting in PF'_{tot} behavior analogy to series model, having near zero PF'_{tot} inside SC2 bandgap. This scenario applies even for the $\alpha = 0.99$ case as shown in Supplementary Figure 12e and g. However, a small peak from PF'_{tot} starts to appear around the first VHS of SC2. Moreover, according to our experimental data, the peak of PF'_{tot} around the first SC2 VHS has to be larger or comparable to the peak around the first SC1 VHS. Therefore, we decided to fix α at 1.

Next, we tuned β while fixing $\alpha = 1$. Supplementary Figure 13b and c show S_{tot} and PF'_{tot} with a β of 0 to 1 varied by 0.1 increments. As shown in Supplementary Figure 13b, the first peak of S around the CNP (marked by *) is suppressed with increasing β, while the second peak around the first VHS of SC2 (marked by †) becomes more dominant. PF'_{tot} also shows two peaks (* and †) between CNP and VHS of SC2 (Supplementary Figure 13c). The first peak (*) arises, which was absent in series case, arises because of finite F_{tot} even withing the bandgap. It gets suppressed with increasing β, while the second peak (†) becomes more dominant.

Supplementary Figure 14 shows S_{tot} and PF'_{tot} as a function of G_{tot} with changing β. The first peak (*) looks sharp becauseG_{tot} is nearly a constant inside the SC1 bandgap (Supplementary Figure 13a). A dip around G_{tot} of around 1×10^{-4} S is not apparent for small β for both S_{tot} and PF'_{tot}.

We chose $\beta = 0.9$ for our model. First, $\beta = 0 - 0.9$ cases satisfy two points which the parallel model failed to reproduce; PF'_{tot} shows overall decrease with G_{tot} in (Supplementary Figure 14b), and both S_{tot} and PF'_{tot} value sharply increase right after the CNP as shown in Supplementary Figure 13f and g. However, the first peak (*) is too dominant with $\beta = 0 - 0.8$ in Supplementary Figure 14 when compared with our gating data (Supplementary Figure 5). Thus, we used $\alpha = 1$ and $\beta = 0.9$ to approximate our DWCNT fiber.

In order to verify our method, we used our model to reproduce results from Ref. [7]. In this study, the authors used two types of SWCNTs, (6,5) semiconducting SWCNT with diameter of 0.76 nm and metallic enriched SWCNTs with the average diameter of 1.40 nm. Their sample 1 (#1) was >99% (6,5), sample 2 (#2) was mixture of 90% (6,5) and 10% metal, sample 3 (#3) was 50% (6,5) and 50% metal, sample 4 (#4) was >99% metal, and sample 5 (#5) was aligned metal. S shows its maximum value around gate voltage of 0 V only for #1, and other samples show maximum S value at higher (#2) or at the highest (#3-5) gate voltage. As shown in Figure 2b of Ref. [7], S shows a sharp value change around gate voltage of 0 V for #1, and #2-#5 also show a finite change although the amplitude is not as large as #1. PF is nearly zero at small gate voltage, and shows a peak (#1 and #2) or increases with gate voltage (#3-5) (Figure 2c of Ref. [7]).

In our model, we used SC1 in Supplementary Table 3 as (6,5) SWCNT and M2 as metallic SWCNTs. We set the ratio of SC1 to M2 as 100:0 for #1, 10:90 for #2, 50:50 for #3, and 0:100 for #4. We set $\alpha = 1$ and $\beta = 0.99$ for #2 and #3.

Supplementary Figure 15a and b show S_{tot} and PF'_{tot}, respectively as a function of chemical potential for sample #1 to #4. Overall, our model adequately reproduces Figure 2 of Ref. [7] qualitatively. S_{ind} shows its maximum value around CNP only for #1, and other samples show peaks around the first VHS. In Figure 2b of Ref. [7], S_{tot} monotonically increased with gate voltage for #3 and 4 because the gated range was not wide enough to obtain the peaks. Note that the tuned chemical potential range differs from sample to sample even when the same gate voltage range was used. PF'_{tot} is nearly zero at and show its peak around first VHS (Supplementary Figure 15). Remarkably, our model was able to reproduce a small jump-like behavior around the charge neutrality point for #2 and 3, by including a small percentage (1%) of the series model. As shown in Supplementary Figure 16, it fails to reproduce this behavior without including the series model ($\beta = 1$).

Supplementary Note 3 Textile Thermoelectric Generator Characterizations

1. Evaluation of flexibility

In order to ensure the performance of the thermoelectric generator (TEG) device while they are bent, we bent one TEG unit (consisting of fifteen CNT threads) with a bending radius of 3.18 mm as shown in Supplementary Figure 18c and d, and applied a temperature difference while maintaining the bending. Supplementary Figure 18e shows a generated voltage as a function of the temperature difference without bending (black) and with bending condition (red). The slope of the two fitting curves differs only by 3.8 %, verifying that there is essentially no degradation in device performance due to bending.

2. Evaluation of output power

From fitting the voltage versus temperature curve of Figure 3d, the slope is 0.00132 V/K. Since there were 60 CNT threads in the device, the Seebeck coefficient of each CNT thread is estimated to be 22.1 μV/K. This value is lower than the highest value in Figure 1c (>60 μV/K) because there was no prior chemical treatment to optimize the Fermi energy position.

Supplementary Figure 21a shows measured voltage drops across the loaded resistors V_{L} (calculated power P_{L}) as a function of measured current I_{tot} on the left (right) axis at each temperature. V_{L}, I_{tot} and P_{L} can be described as

$$I_{\mathrm{tot}} = \frac{V_{\mathrm{tot}}}{R_{\mathrm{CNT}} + R_{\mathrm{L}}} \tag{13}$$

$$V_{\mathrm{L}} = R_{\mathrm{L}} \times \frac{V_{\mathrm{tot}}}{R_{\mathrm{CNT}} + R_{\mathrm{L}}}, \tag{14}$$

$$P_{\mathrm{L}} = V_{\mathrm{L}} \times I_{\mathrm{tot}} \tag{15}$$

where V_{tot} is the total voltage generated by the thermoelectric generator, R_{CNT} is the resistance of the thermoelectric generator, and R_{L} is the resistance of the loaded resistor. V_{tot} is defined as $V_{\mathrm{tot}} = S \times \Delta T \times N$, where S is the Seebeck coefficient of each CNT thread (=22.1 μV/K in this case as described above), ΔT is the temperature difference applied, and N is the number of CNT threads ($N = 60$). We fit V_{L} versus I_{tot} using Supplementary Equations (13) and (14) with R_{CNT} as a fitting parameter. We allowed R_{CNT} to change with the temperature. As shown in Supplementary Figure 21a, the fitting and the measured values show excellent agreement. Supplementary Figure 21b shows P_{L} as a function of R_{L} at ΔT of 62.5 K to show that P_{L} is maximized when $R_{\mathrm{L}} = R_{\mathrm{CNT}}$.

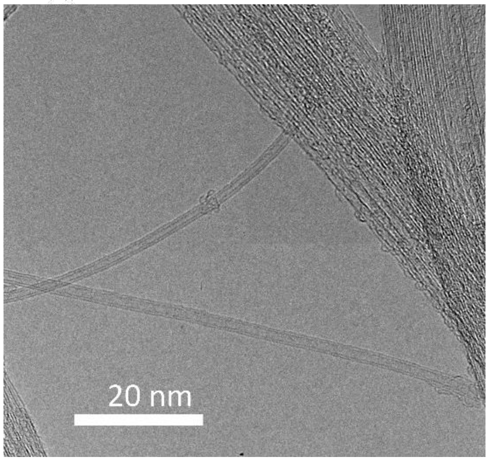

Supplementary Figure 1. High resolution transmission electron microscopy image of the raw CNT material. The average outer (inner) wall diameter was determined to be 1.8±0.2nm (0.9±0.1 nm) and the average number of walls was 1.9.

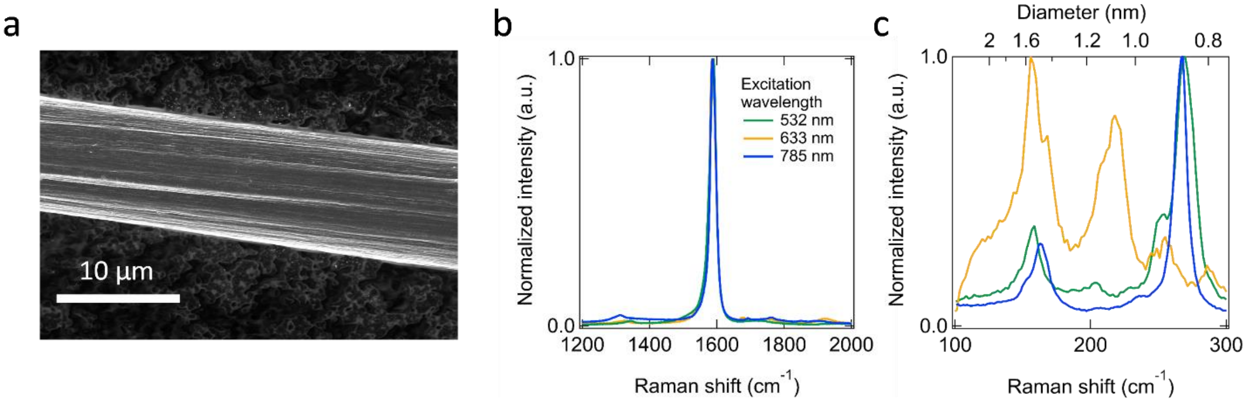

Supplementary Figure 2. Characterization of as-produced CNT fibers. (a) SEM image of the surface morphology of the fiber produced by solution spinning method, depicting highly aligned CNT bundles. The average diameter was measured to be 8.9±0.9 µm. (b) Raman spectra for the as-produced CNT fibers excited by 532 nm (green), 633 nm (yellow), and 785 nm (blue) lasers. The average G-to-D ratio was determined to be 50, 45, and 25 for the 532 nm, 633 nm, and 785 nm excitations. (c) Raman spectra in the radial breathing mode region. Raman shift was converted into the diameter using the relationship proposed in Ref. [13].

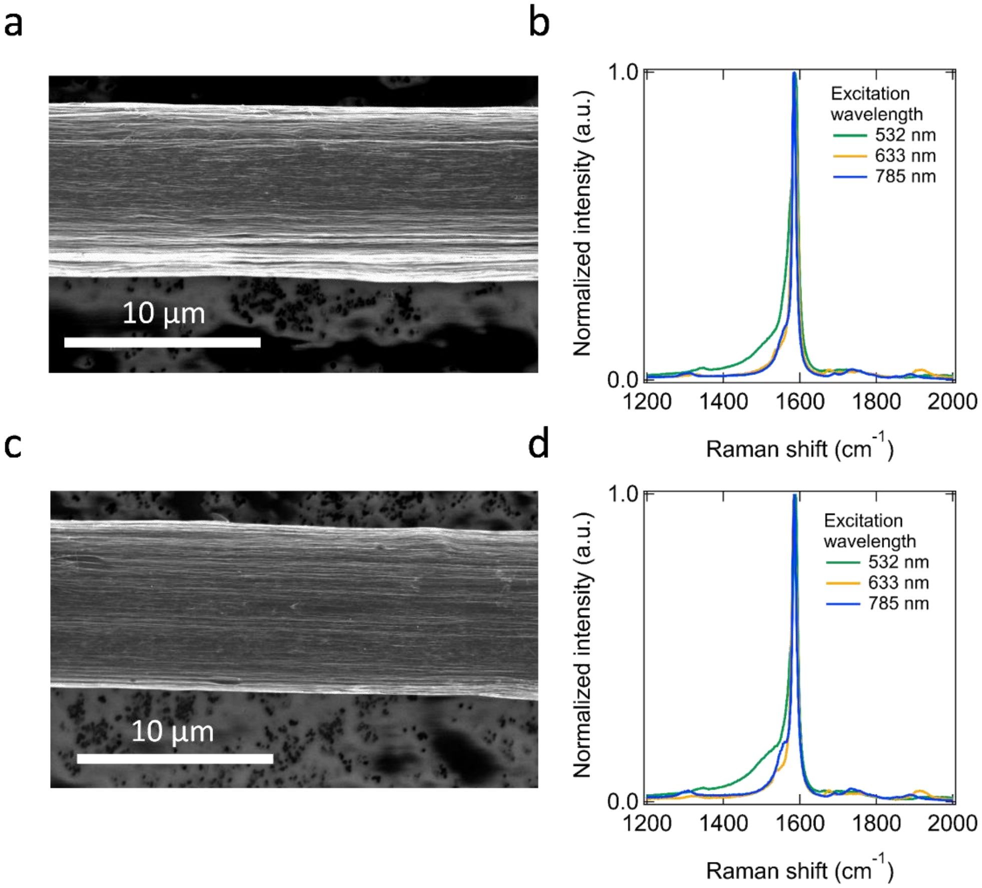

Supplementary Figure 3. Characterization of CNT fibers annealed at (a) & (b) 350 ˚C and (c) & (d) 500 ˚C. (a) SEM image of the fiber annealed at 350 ˚C. (b) Raman spectra for the fiber annealed at 350 ˚C excited by 532 nm (green), 633 nm (yellow), and 785 nm (blue) lasers. The average G-to-D ratio was determined to be 24, 49, and 40 for the 532 nm, 633 nm, and 785 nm excitation wavelengths, respectively. The decrease in G-to-D ratio compared to the as-produced fibers is mainly attributed to shoulders of the G band giving higher background, not to induced defects. (c) SEM image of the fiber annealed at 500 ˚C. (d) Raman spectra for the fibers annealed at 500 ˚C. The average G-to-D ratio was determined to be 20, 48, and 29 for the 532 nm, 633 nm, and 785 nm excitation wavelengths, respectively.

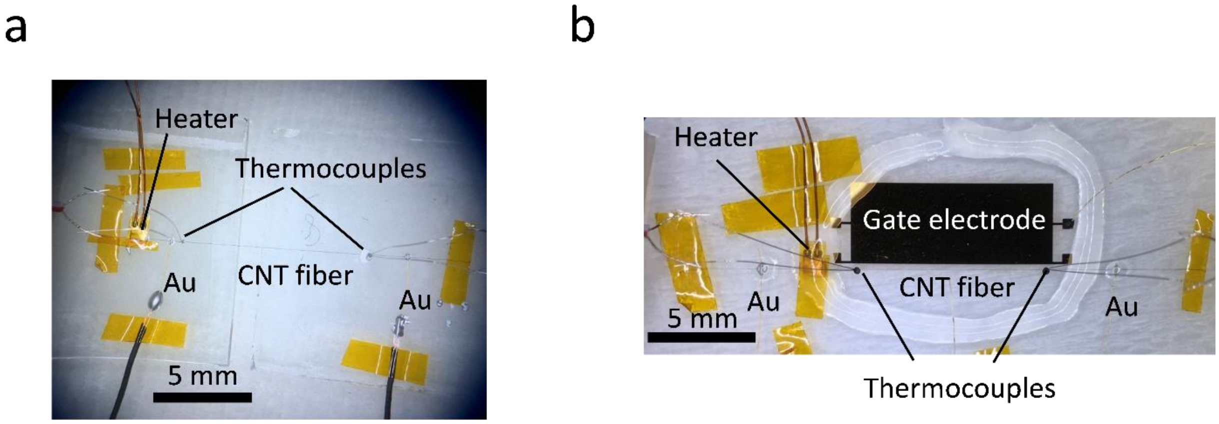

Supplementary Figure 4. (a) Picture of a device used on a chemically treated sample without gating. (b) Picture of a device used for the gating measurement.

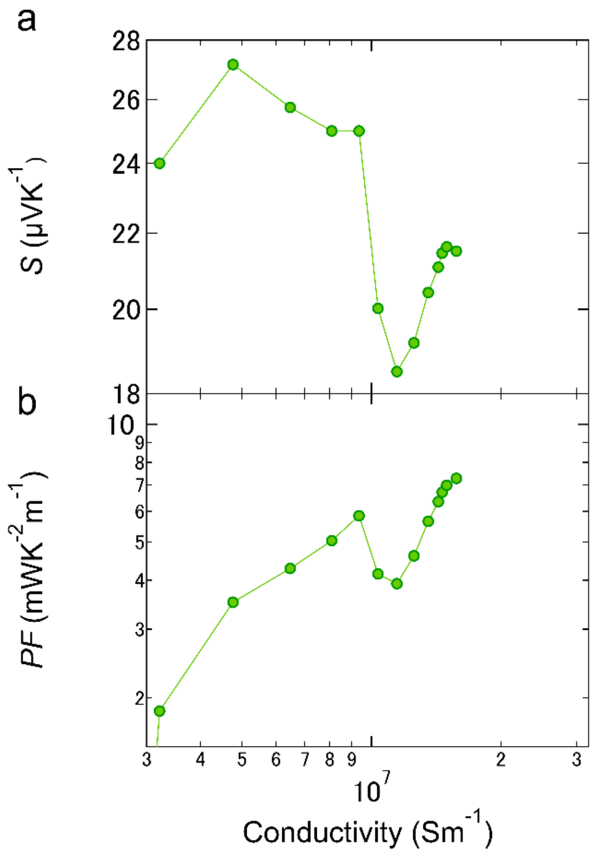

Supplementary Figure 5. (a) S and (b) PF of p-side from a gating experiment as a function of measured electrical conductivity.

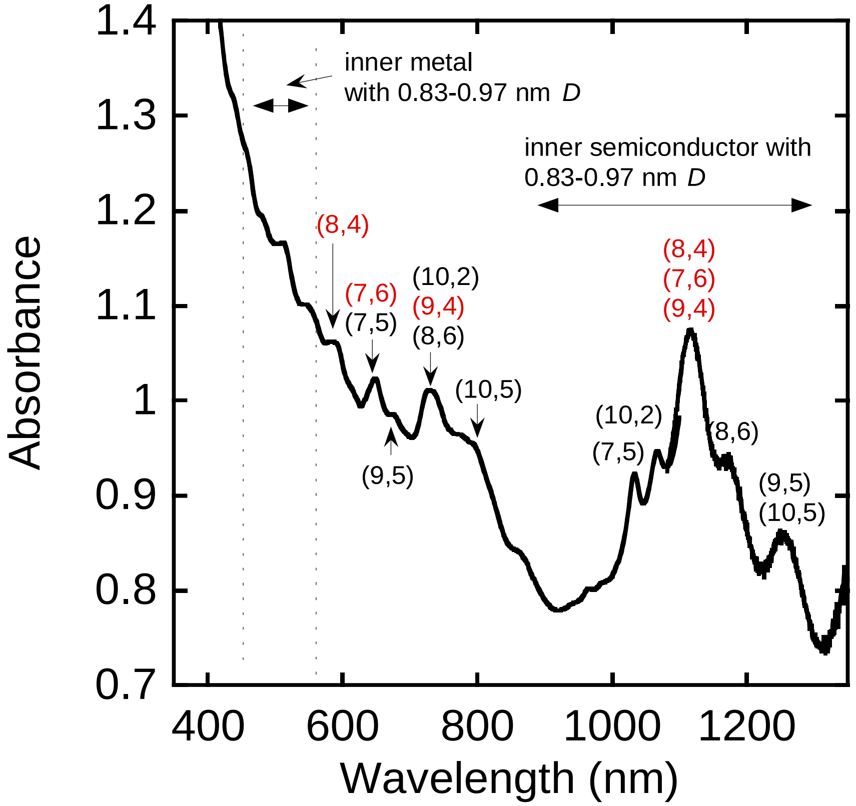

Supplementary Figure 6. Absorbance spectrum of the solution of the raw CNT material. Chirality of inner wall nanotubes were assigned based on Ref [2,3].

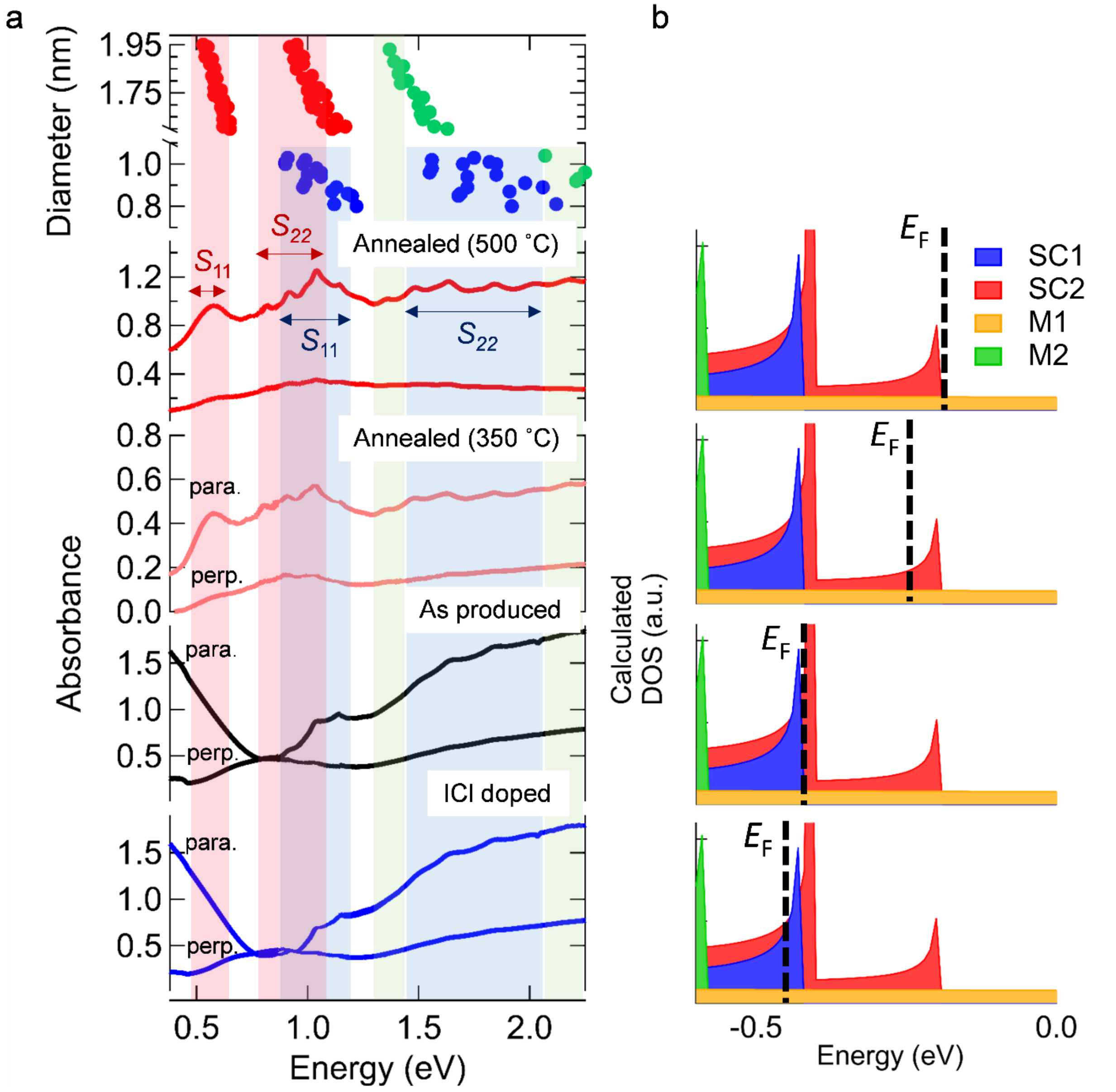

Supplementary Figure 7. Optical studies of CNTs aligned films. (a) (Top) Expected absorbance peak positions based on diameter information from Ref [2,3], including transition peaks due to the outer semiconducting tubes (marked as S_{11} and S_{22}, red), inner semiconducting tubes (blue), and outer and inner metallic tubes (M_{11}, green). (Bottom) Absorbance spectra of CNT films with the same chemical treatments as Figures 1c and d; annealing at 500 ˚C, annealing at 350 ˚C, as produced, and ICl doping. For each sample, both parallel (when the incident light polarization was parallel to the CNT alignment direction) and perpendicular (when the incident light polarization was perpendicular to the CNT alignment direction) are shown. For the perpendicular case of as produced and ICl doped samples, a new peak appears around 0.8 eV, considered to be the inter subband plasmon peak [4]. (b) Calculated density of states (DOS) as a function of energy, for SC1, SC2, M1, and M2 (see Supplementary Table 3 for their definition.) The expected E_F position deduced from analysis of the spectra in (a) is shown by the black dashed line in each case.

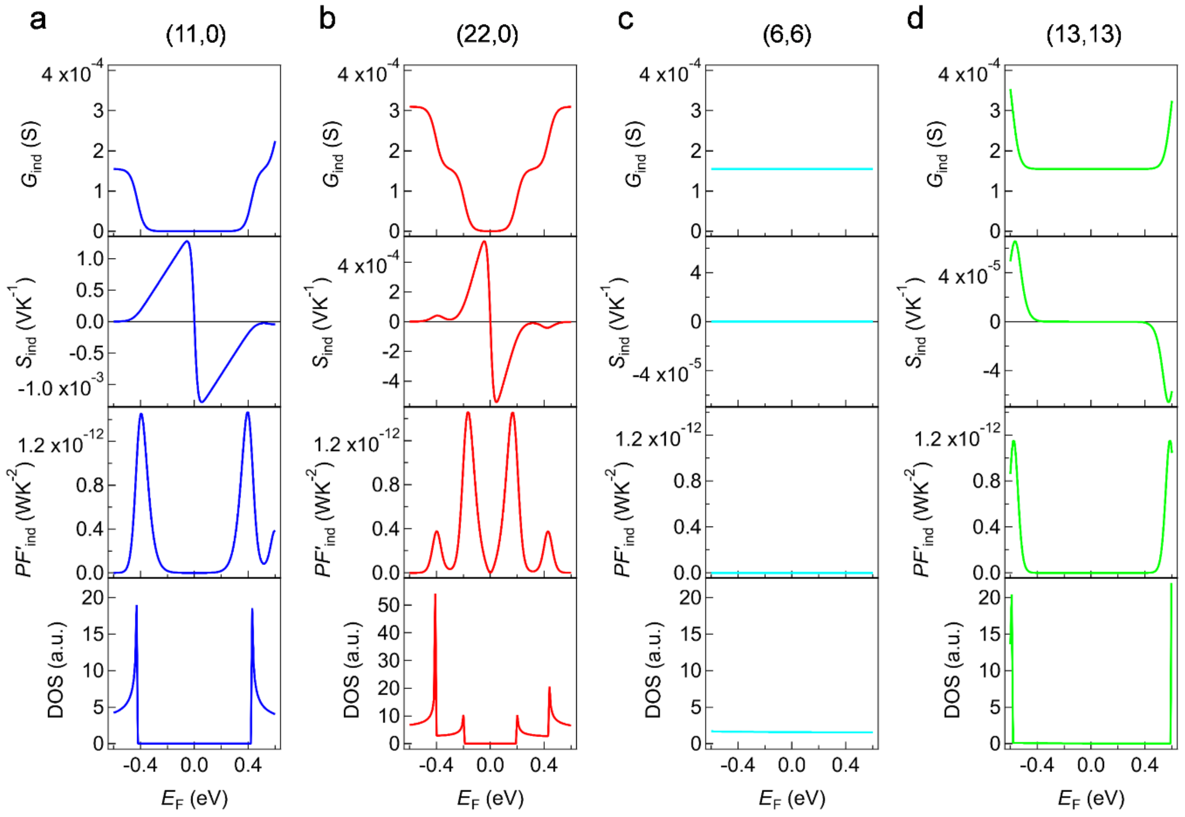

Supplementary Figure 8. Calculated G_{ind}, S_{ind}, and PF'_{ind} for four representative SWCNTs, (a) SC1 (11,0), (b) SC2 (22,0), (c) M1 (6,6) and (d) M2 (13,13).

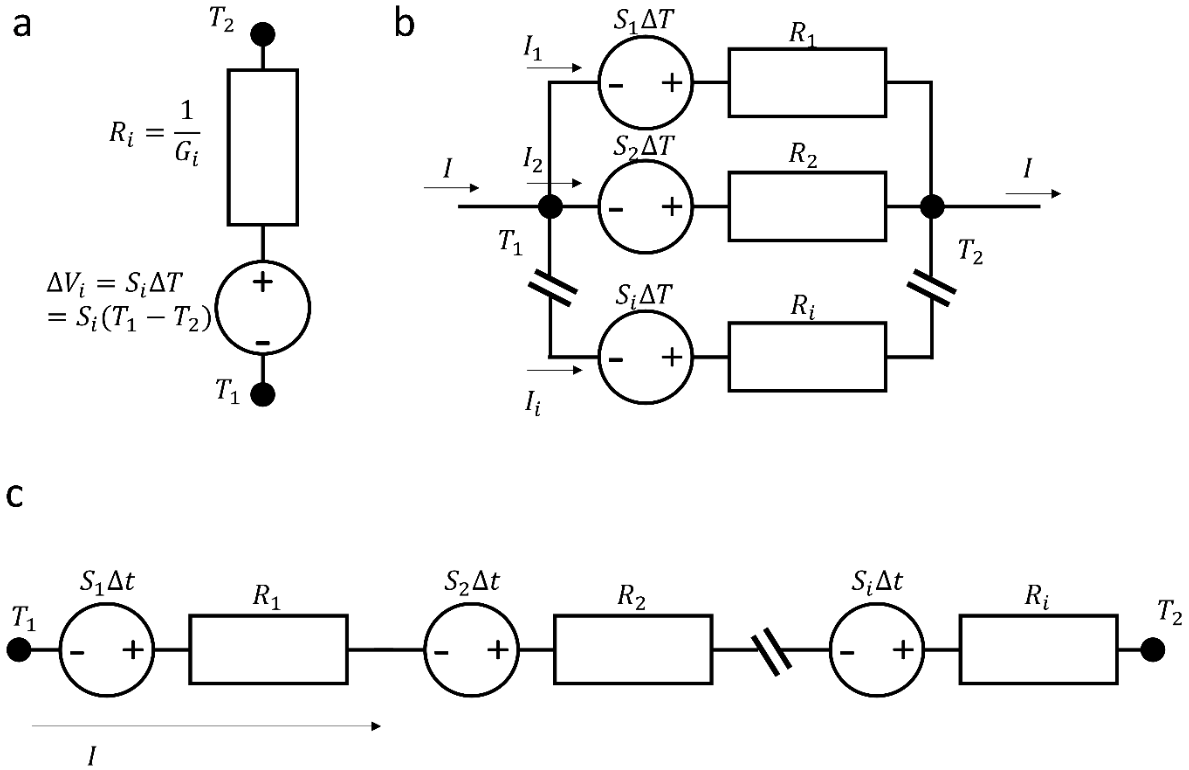

Supplementary Figure 9. Circuit models used to approximate the DWCNT system. (a) Each SWCNT is approximated by an equivalent circuit components of a generator ($S_i\Delta T$) and a resistor ($R_i = 1/G_i$), where i denotes that this is the ith nanotube in the system. (b) Nanotubes described in (a) are connected in parallel. A temperature gradient applied per one nanotube ΔT is assumed to be the same for all nanotubes for simplicity. (c) Nanotubes described in (a) are connected in series. A temperature gradient applied per one nanotube Δt is assumed to be the same for all nanotubes.

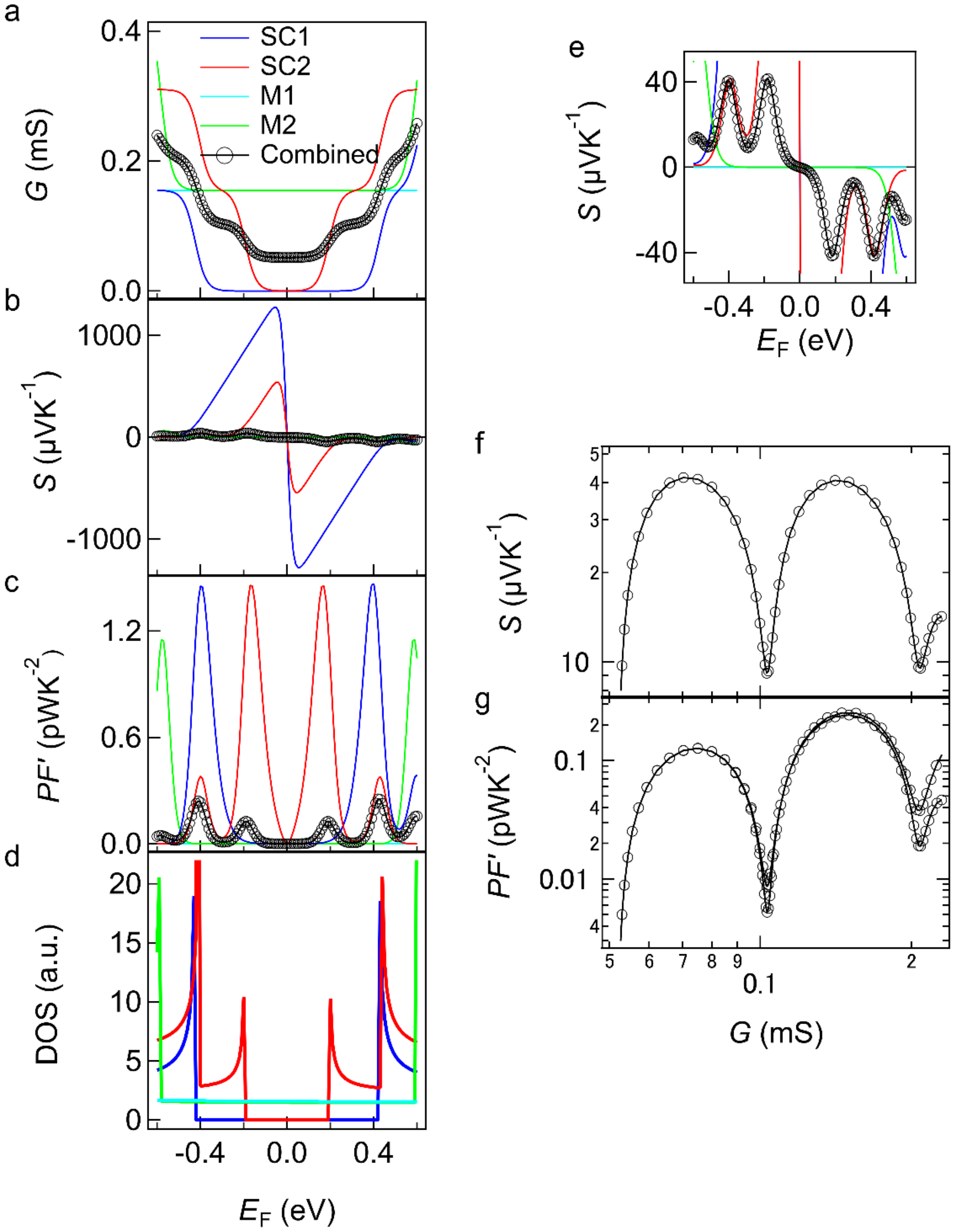

Supplementary Figure 10. Calculated results using a parallel circuit model using Supplementary Equations (4) and (5). Calculated (a) G_{p}, (b) S_{p}, and (c) PF'_{p}combining four SWCNTs using a parallel model as a function of chemical potential shown in a black line with open circles. G_{ind}, S_{ind}, and PF'_{ind} of SC1 (blue), SC2 (red), M1 (cyan), and M2 (green) from Supplementary Figure 8 are shown for a comparison. (d) Density of states of SC1 (blue), SC2 (red), M1 (cyan), and M2 (green). (e) Magnified image of Supplementary Figure 10b. (f) S_{p} and (g) PF'_{p} of combined result as a function of G_{p}.

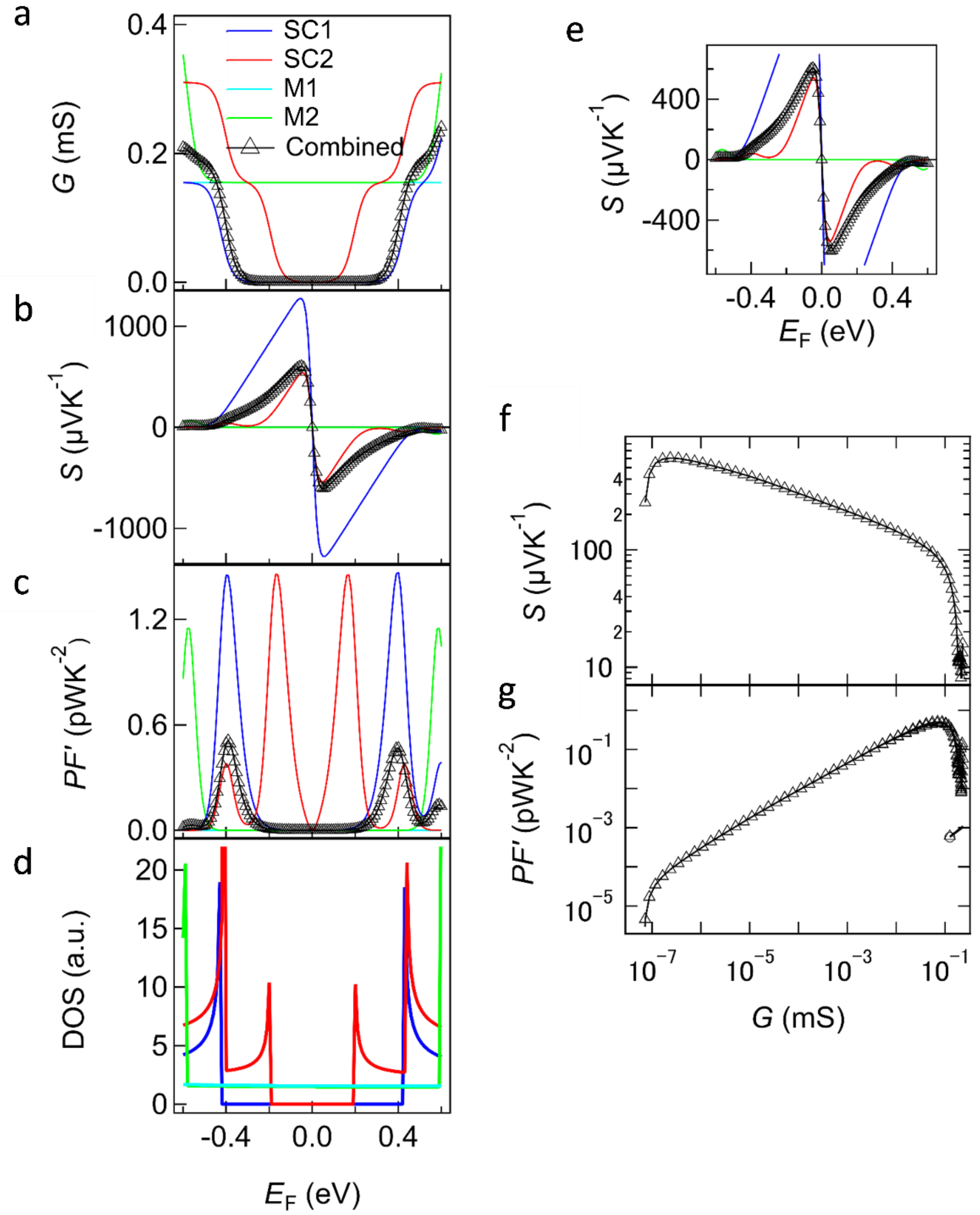

Supplementary Figure 11. Calculated results using a series circuit model using Supplementary Equations (7) and (8). Calculated (a) G_s, (b) S_s , and (c) PF'_s combining four SWCNTs using a series model as a function of chemical potential shown in a black line with open circles. G_{ind}, S_{ind}, and PF'_{ind} of SC1 (blue), SC2 (red), M1 (cyan), and M2 (green) from Supplementary Figure 8 are shown for a comparison. (d) Density of states of SC1 (blue), SC2 (red), M1 (cyan), and M2 (green). (e) Magnified image of Supplementary Figure 11b. (f) S_s and (g) PF'_s of combined result as a function of G_s.

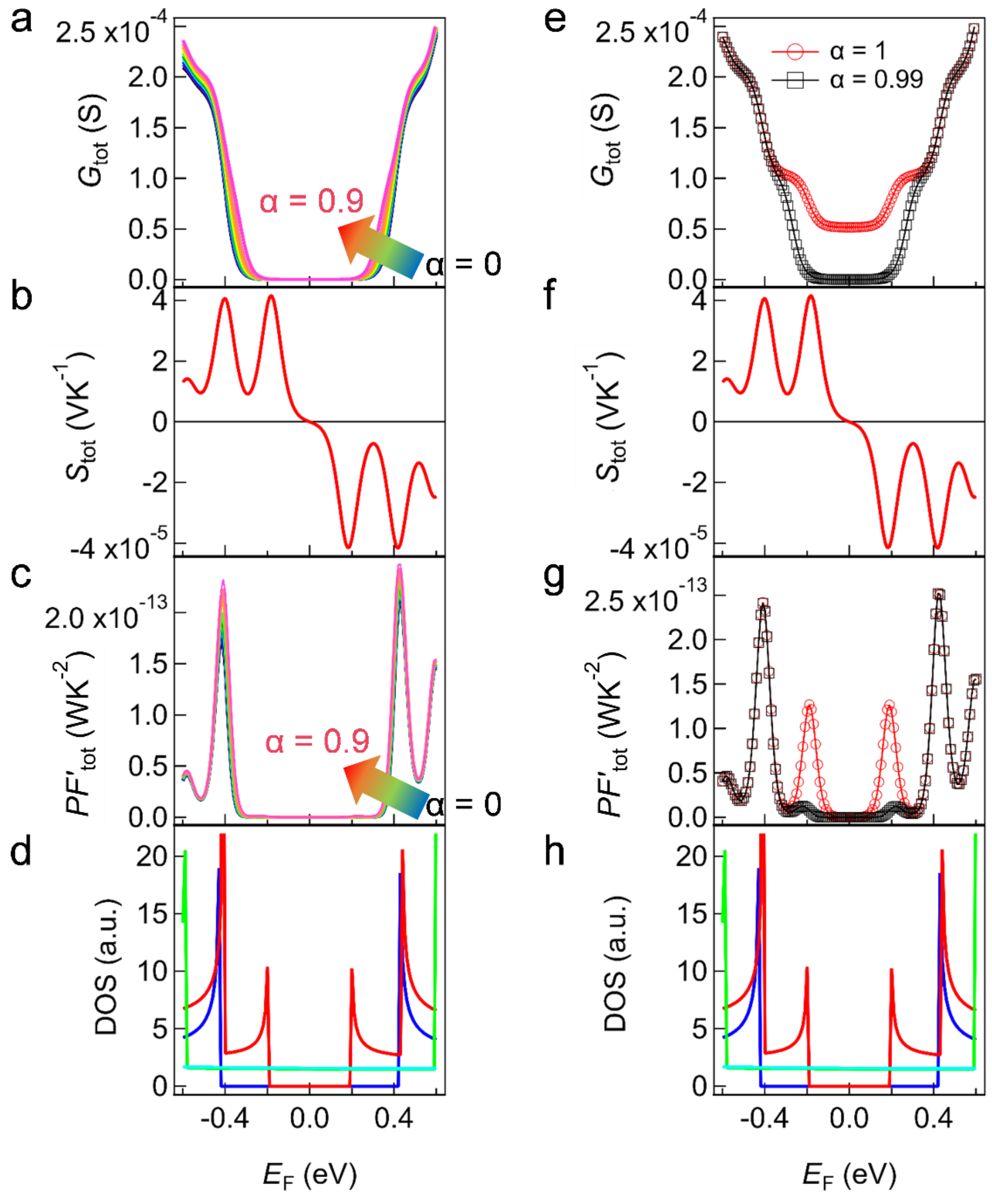

Supplementary Figure 12. Tuning α in Supplementary Equation (12). Calculated (a) G_{tot}, (b) S_{tot}, and (c) PF'_{tot} as a function of chemical potential with α of 0 to 0.9 by 0.1 while fixing $\beta = 1$. (d) Density of states of SC1 (blue), SC2 (red), M1 (cyan), and M2 (green). Calculated (e) G_{tot}, (f) S_{tot}, and (g) PF'_{tot} as a function of chemical potential with α of 0.99 and 1 while fixing $\beta = 1$. (h) Density of states as a reference.

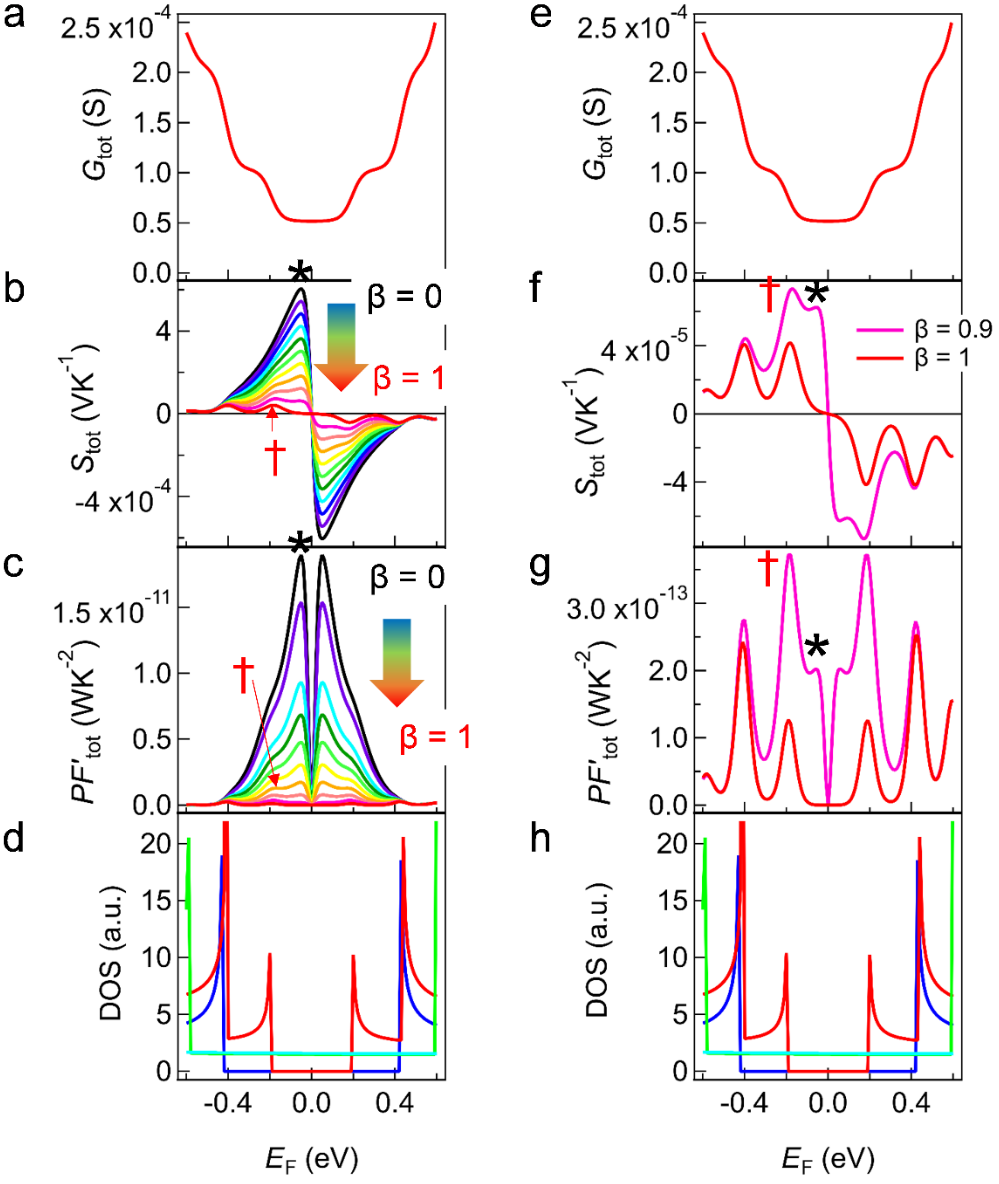

Supplementary Figure 13. Tuning β in Supplementary Equation (11). Calculated (a) G_{tot} , (b) S_{tot}, and (c) PF'_{tot} as a function of chemical potential with β of 0 to 1 by 0.1 while fixing $\alpha = 1$. (d) Density of states of SC1 (blue), SC2 (red), M1 (cyan), and M2 (green). Calculated (e) G_{tot}, (f) S_{tot}, and (g) PF'_{tot} as a function of chemical potential with β of 0.9 and 1 while fixing $\alpha = 1$. (h) Density of states as a reference.

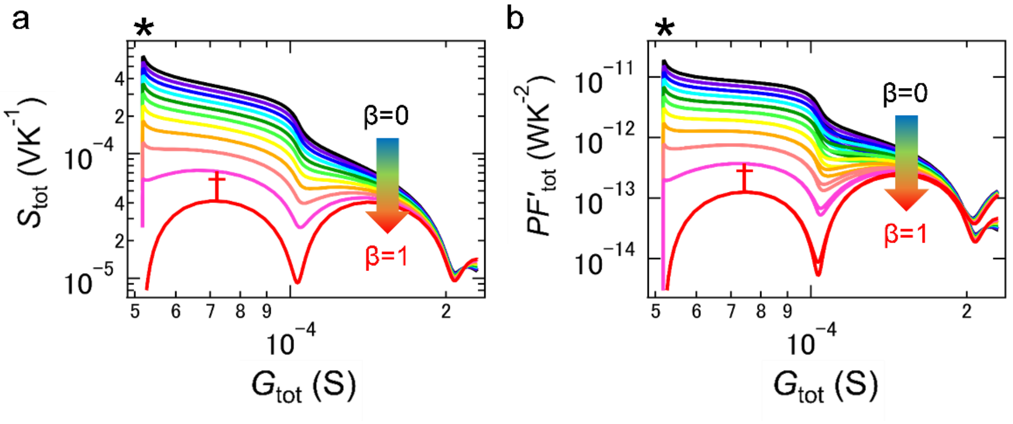

Supplementary Figure 14. (a) S_{tot} and (b) PF'_{tot} as a function of G_{tot} with β of 0 to 1 by 0.1 increments while fixing $\alpha = 1$ from Supplementary Equation (11).

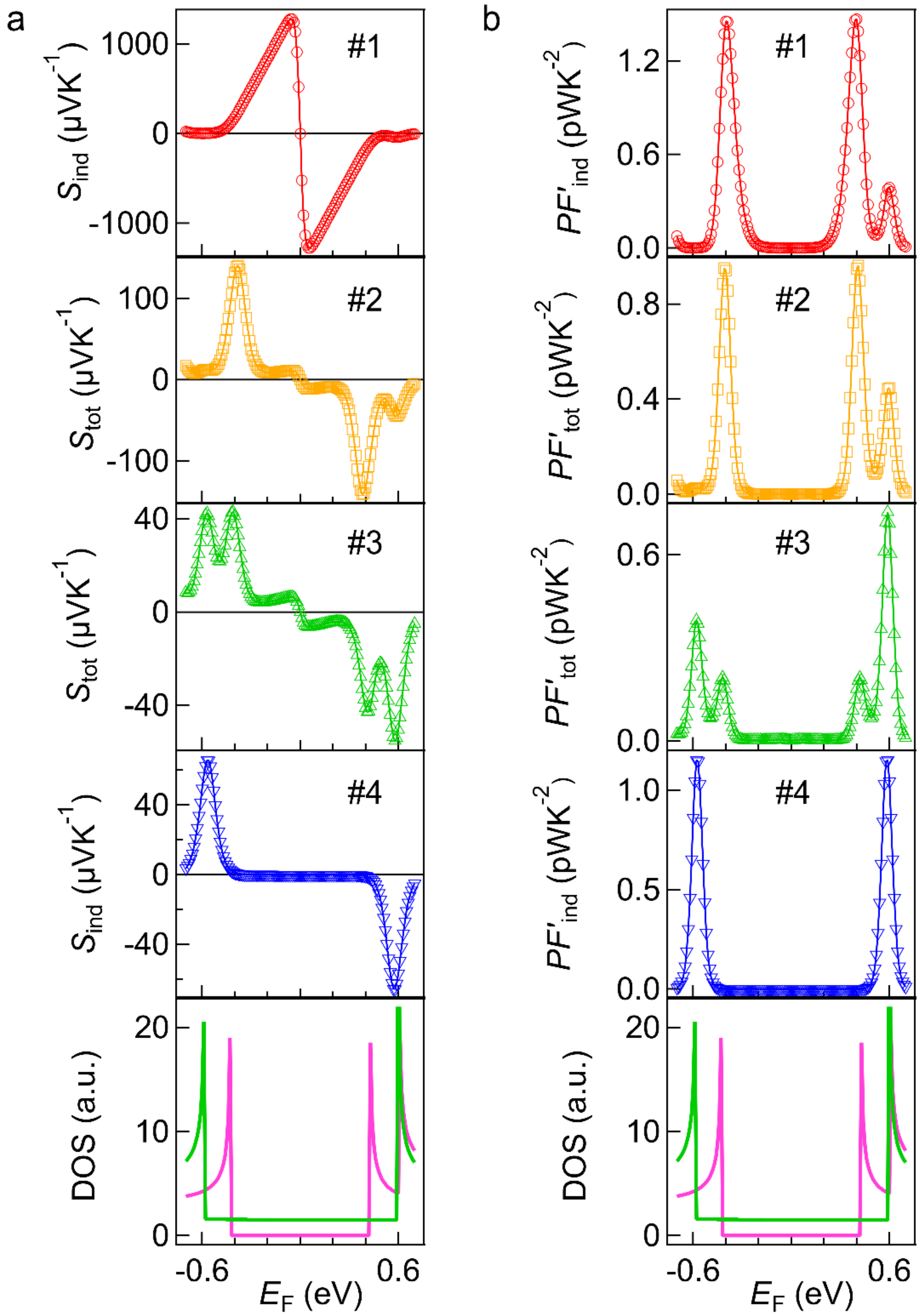

Supplementary Figure 15. Calculated (a) S_{tot}, and (b) PF'_{tot} as a function of chemical potential. #1 is the same as SC2 in Supplementary Figure 8 and #4 is the same as M1 in Supplementary Figure 8. For #2 and #3, we used the parallel and series combined model with $\beta = 0.99$ (Supplementary Equation (11)) and $\alpha = 1$ from Supplementary Equation (12). The ratio of SC2:M1 is 90:10 for #2 and 50:50 for #3. DOS of SC2 and M1 is shown as a reference.

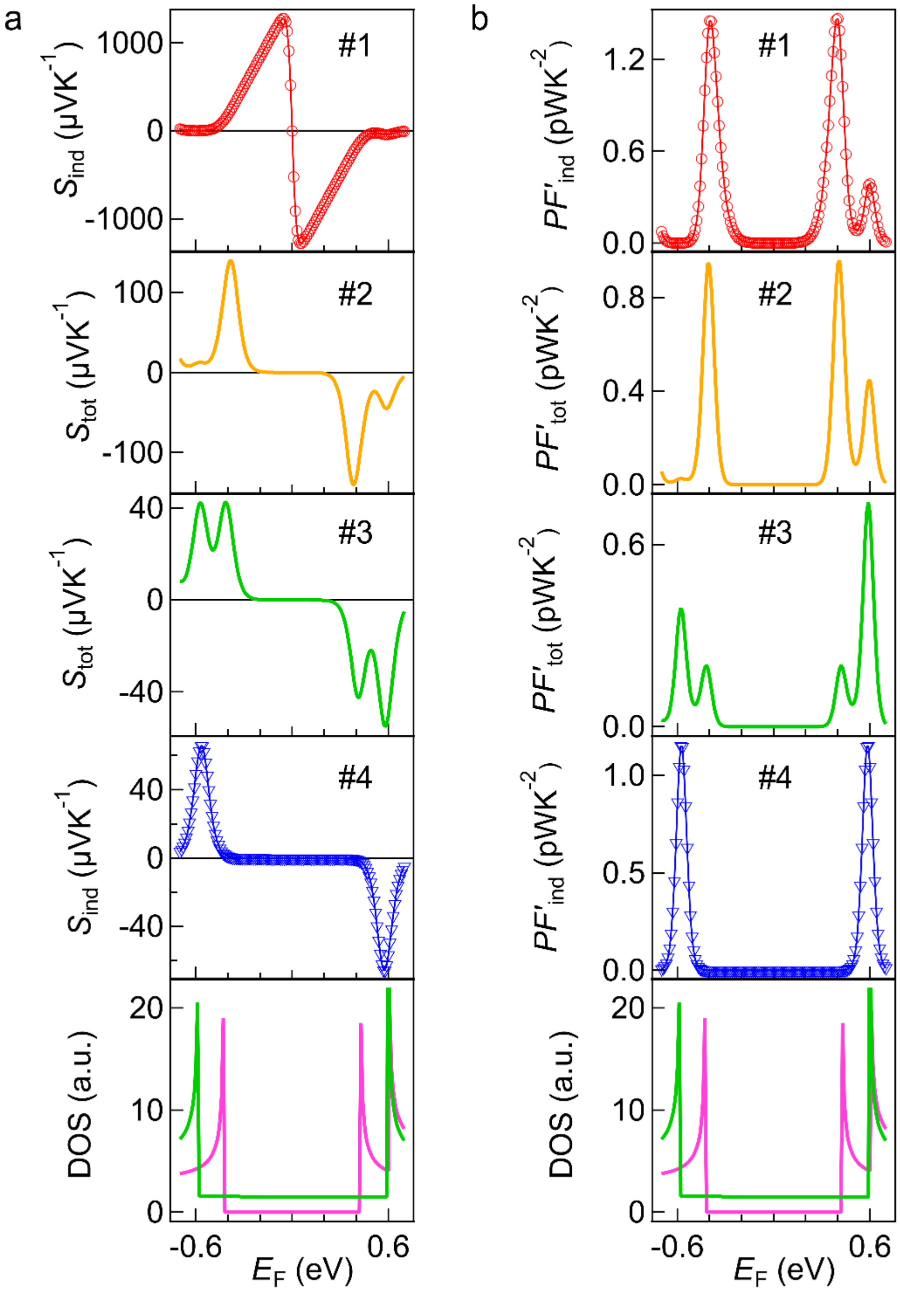

Supplementary Figure 16. Calculated (a) S_{tot}, and (b) PF'_{tot} as a function of chemical potential. #1 is the same as SC2 in Supplementary Figure 8 and #4 is the same as M1 in Supplementary Figure 8. For #2 and #3, we used the parallel and series combined model with $\beta = 1$ (Supplementary Equation (11)) and $\alpha = 1$ from Supplementary Equation (12). The ratio of SC2:M1 is 90:10 for #2 and 50:50 for #3. DOS of SC2 and M1 is shown as a reference.

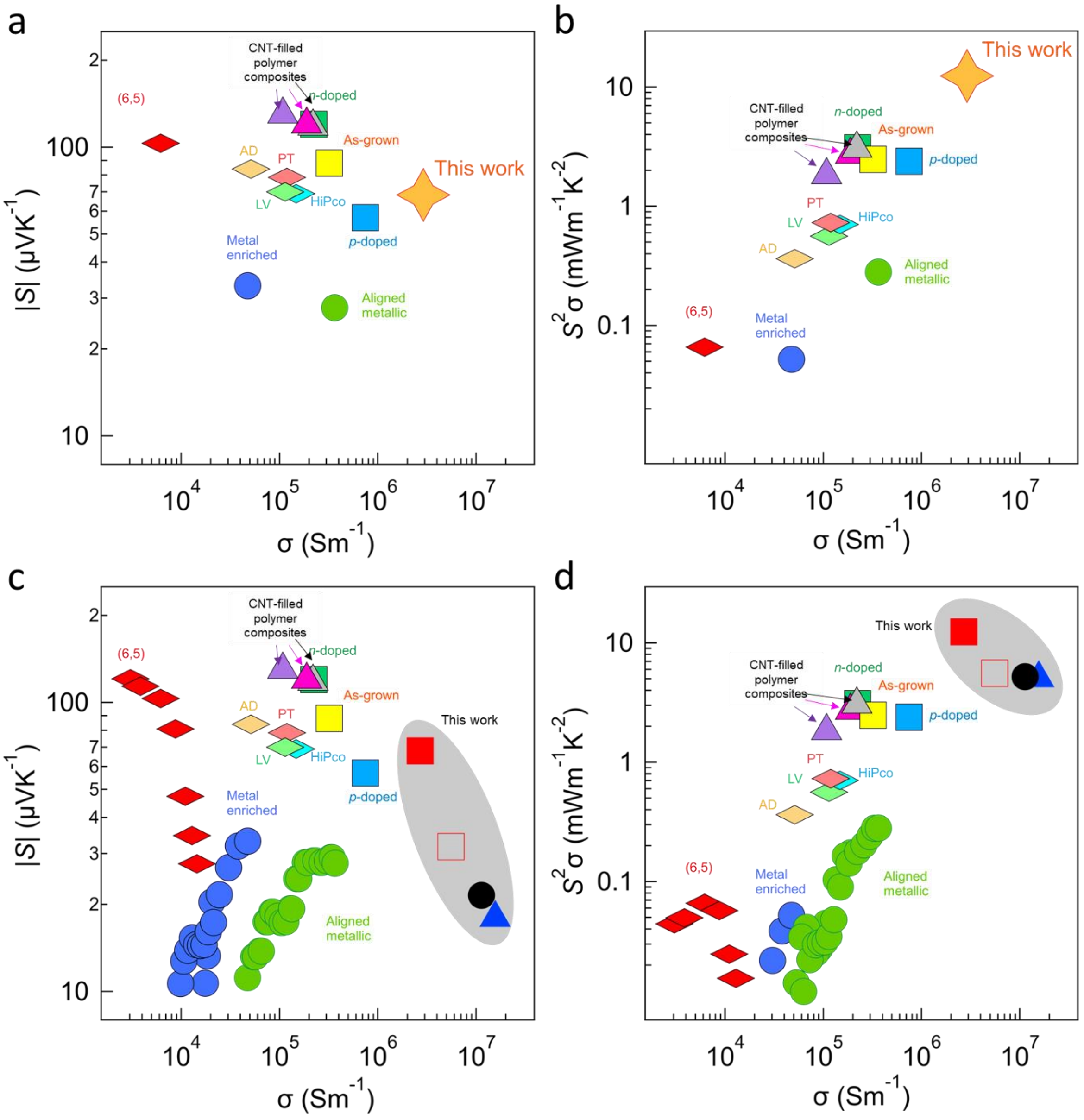

Supplementary Figure 17. Comparison of reported (a) Seebeck coefficient and (b) power factor for various CNT samples as a function of electrical conductivity. (b) Figure 1e without non-CNT references. Values are summarized in Supplementary Table 4. (c) Seebeck coefficient and (d) power factor with varying electrical conductivity for this work, (6,5) SWCNTs, metal enriched SWCNTs and aligned metallic SWCNTs.

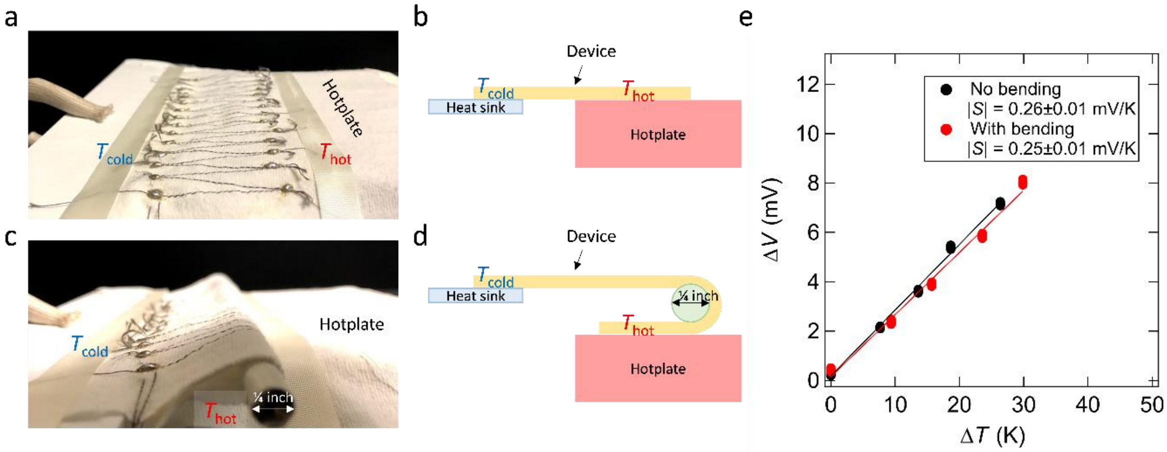

Supplementary Figure 18. (a) No bending condition, where one TEG unit (consisting of fifteen CNT threads) was suspended between a hotplate and a heat sink. The temperature difference was created by increasing the hot side temperature (T_{hot}) by the hotplate, while no modification was added to the cold side (T_{cold}). (b) Schematic image of (a). (c) Bending condition, where the device was bent with a bending radius of 3.18 mm. The hot side was directly underneath the inserted bar. This position (with the bending radius of 3.18 mm) was fixed throughout the temperature dependence of generated voltage measurement. (d) Schematic image of (c). (e) Generated voltage from one TEG unit as a function of the temperature difference T_{hot}- T_{cold} with no bending (black) and with bending (red). The fitting gives the total Seebeck coefficient for one TEG unit of 0.26±0.01 mV/K for no bending and that of 0.25±0.01 mV/K with bending.

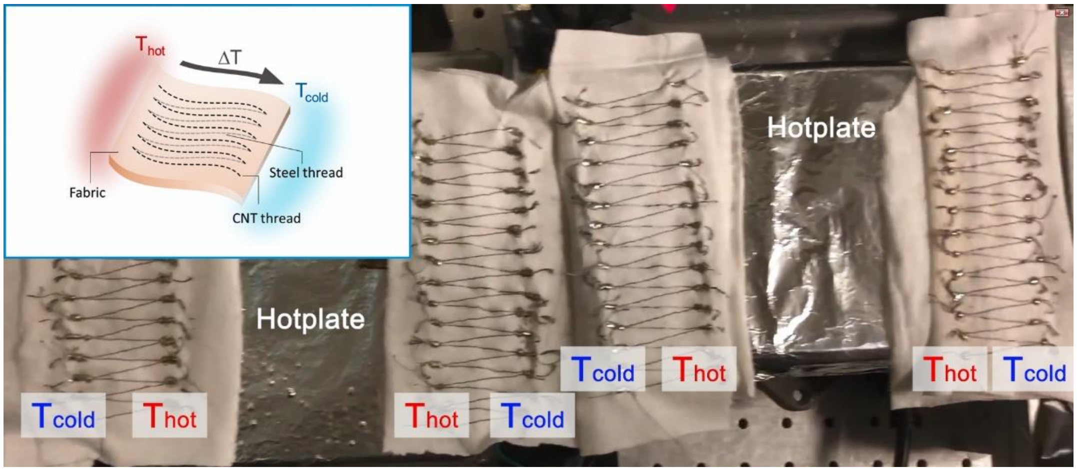

Supplementary Figure 19. The CNT thread and steel thread were sewn into a fabric. Fifteen CNT threads were connected thermally in parallel and electrically in series to create one TEG unit, and the four units were connected in series. The cold side was at room temperature, and the hot side was placed on hotplates. The inset shows the schematic image of a textile TEG.

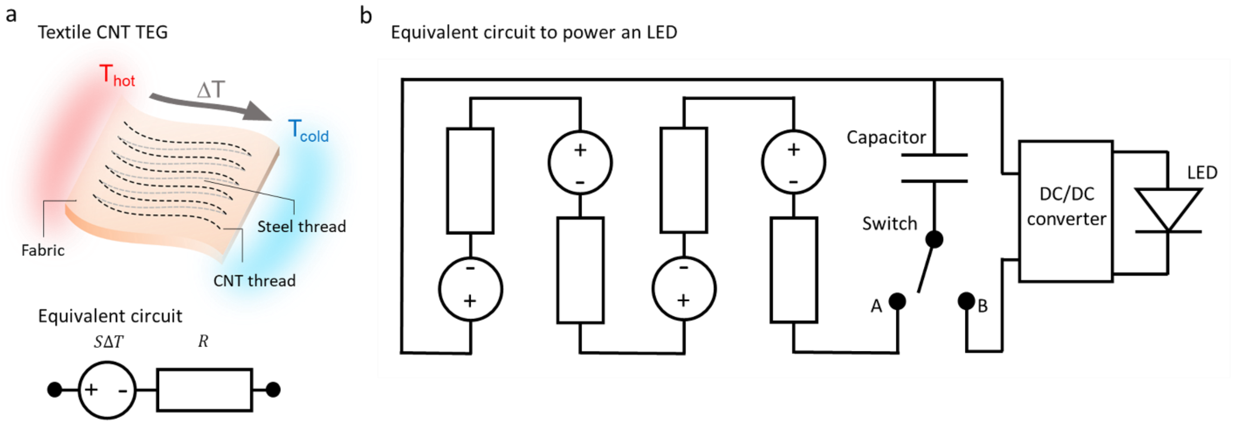

Supplementary Figure 20. (a) One TEG unit is equivalent to a voltage source and a resistor. (b) The entire circuit to power the LED consists of four TEG units connected in series, a capacitor, a DC/DC converter, an LED, and a switch. The converter operates when the input voltage is 40 mV or higher, and the output voltage is 1 to 10 V depending on input voltage and load. When the switch is connected to A, the capacitor is charged by the TEGs. When it switches to B, the capacitor discharges and lights up the LED.

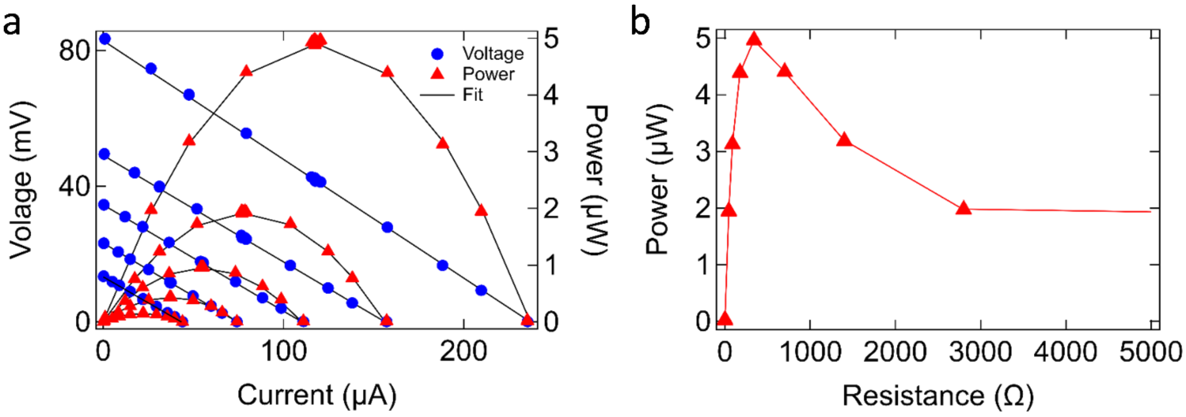

Supplementary Figure 21. (a) The output voltage and power at temperature differences, fitted by Supplementary Equations (13)-(15). (b) The output power as a function of loaded resistance at a temperature difference of 62.5 K.

Supplementary Table 1. Dimensions of CNTs, CNT fibers, and CNT threads used in this study.

		CNTs	CNT fibers	CNT threads
For thermoelectric study	Production source	Meijo NC	Solution spinning CNTs	
	Average diameter	1.8 nm	8.9 µm	
	Length	12 µm (average)	> 100 m	
For TEG demonstration	Production source	Meijo NC		Plying 21 CNT filaments together
	Average diameter	1.8 nm		190 µm
	Length	7.4 µm (average)		> 100 m

Supplementary Table 2. Four types of samples measured with different chemical treatments

Chemical treatment	Electrical conductivity (MS/m)	Seebeck coefficient (µV/K)	Power factor (mW/mK^2)
ICl doped	15±3	18.0±0.2	5±1
N/A (as produced)	11±2	21.5±0.2	5±1
Annealed at 350 ˚C	5.6±1.1	31.7±0.2	6±1
Annealed at 500 ˚C	2.7±0.5	68.0±0.3	12±2

Supplementary Table 3. Four representative SWCNTs used to describe DWCNT fiber

	Chirality	Diameter (nm)	Electronic type
SC1	(11,0)	0.87	Semiconducting
SC2	(22,0)	1.75	Semiconducting
M1	(6,6)	0.80	Metallic
M2	(13,13)	1.73	Metallic

Supplementary Table 4. Comparison of reported *PF* values for various CNT samples.

	Sample details (original production method)	Dopant	σ (S/m)	S (μV/K)	PF (mW/mK^2)	Reference
Unsorted	Buckypaper (CVD)	N/A (no intentional doping)	3.2×10^5	88	2.48	[14]
	Aligned yarn (CVD)	$FeCl_3$	7.5×10^5	57	2.39	[15]
	Web (CVD)	BV	2.2×10^5	-120	3.10	[16]
	Aligned fiber (eDIPS)	N/A (annealed)	2.9×10^6	68	14	This work
Semiconductor-enriched	Film (AD)	OA	5.1×10^4	84	0.36	[17]
	Film (pulsed LV)		1.1×10^5	70	0.56	
	Film (HiPco)		1.5×10^5	69	0.71	
	Film (Plasma Torch)	BV	1.2×10^5	-79	0.73	
	(6,5) film (HiPco)	N/A (no intentional doping)	6.2×10^3	103	0.066	[7]
Metal-enriched	Film (AD)		4.8×10^4	33	0.052	
	Aligned film (AD)		3.6×10^5	28	0.28	
CNT-filled polymer nanocomposites	PANI/graphene/PANI/DWCNT	N/A (no intentional doping)	1.1×10^5	130	1.83	[18]
	PANI/graphene-PEDOT:PSS/PANI/DWCNT-PEDOT:PSS		1.9×10^5	120	2.71	[19]

	PANI/graphene/PANI/DWCNT		2.2×10^5	118	3.05	[20]

*Abbreviations used; CVD: chemical vapor deposition, eDIPS: enhanced direct injection pyrolytic synthesis, BV: benzyl viologen, AD: arc-discharge, LV: laser vaporization, OA: Triethyloxonium hexachloroantimonate, PANI: polyaniline, and PEDOT:PSS: Poly(3,4ethylenedioxythiophene):poly(styrenesulfonate)

Supplementary Table 5. Comparison of calculated effective thermal conductivity κ_{eff}, defined as[21] $\kappa_{\text{eff}} = \kappa + \frac{PFT^2}{2\Delta T}$, for representative materials at a ΔT of 1 K.

Materials		T (K)	σ (S/m)	S (μV/K)	PF (mW/mK^2)	κ (W/mK)	κ_{eff} (W/mk)	Ref.
	CNT fiber	300	2.9×10^6	68	14	580*	1,190	This work
Metals	Co	300	1.7×10^7	-30	15	100	780	[22]
	$YbAl_3$	300	2.7×10^6	-77	16	21	740	[23]
	CuNi	400	2.1×10^6	-53	5.8	29	460	[24]
	AgPd	400	3.2×10^6	-40	5.1	36	440	[24]
	$CePd_3$	300	9.1×10^5	92	7.7	10	360	[25]
	Alumel	300	3.4×10^6	-16	0.8	29	70	[26,27]
	Chromel	300	1.4×10^6	26	1.0	17	60	[26,27]
Conventional TE	Bi_2Te_3	300	1.0×10^5	210	4.5	1.2	200	[28]
	$Cu_{0.9}Ni_{0.1}AgSe$	300	5.9×10^5	-85	5.8	4.7	200	[29]
	$Mg_3Bi_{1.25}Sb$	300	9.1×10^4	-210	4.1	1.5	180	[30]

materials	$CsBi_4Te_6$	300	1.0×10^5	105	1.1	0.5	50	[31]

*Thermal conductivity value was taken from Ref. [32]

Supplementary References

1. Headrick, R. J. *et al.* Structure–Property Relations in Carbon Nanotube Fibers by Downscaling Solution Processing. *Adv. Mater.* **30**, 1704482 (2018).
2. Weisman, R. B. & Bachilo, S. M. Dependence of optical transition energies on structure for single-walled carbon nanotubes in aqueous suspension: An empirical Kataura plot. *Nano Lett.* **3**, 1235–1238 (2003).
3. Weisman, B. R. & Kono, J. *Optical Properties of Carbon Nanotubes: A Volume Dedicated to the Memory of Professor Mildred Dresselhaus*. (World Scientific Publishing, 2019).
4. Yanagi, K. *et al.* Intersubband plasmons in the quantum limit in gated and aligned carbon nanotubes. *Nat. Commun.* **9**, 1121 (2018).
5. Koskinen, P. & Mäkinen, V. Density-functional tight-binding for beginners. *Comput. Mater. Sci.* **47**, 237–253 (2009).
6. Stokbro, K. *et al.* Semiempirical model for nanoscale device simulations. *Phys. Rev. B* **82**, 075420 (2010).
7. Ichinose, Y. *et al.* Solving the Thermoelectric Trade-Off Problem with Metallic Carbon Nanotubes. *Nano Lett.* **19**, 7370–7376 (2019).
8. Hayashi, D. *et al.* Thermoelectric properties of single-wall carbon nanotube networks. *Jpn. J. Appl. Phys.* **58**, 075003 (2019).
9. Romero, H. E., Sumanasekera, G. U., Mahan, G. D. & Eklund, P. C. Thermoelectric power of single-walled carbon nanotube films. *Phys. Rev. B* **65**, 1–6 (2002).
10. Esfarjani, K. & Zebarjadi, M. Thermoelectric properties of a nanocontact made of two-capped single-wall carbon nanotubes calculated within the tight-binding approximation. *Phys. Rev. B* **73**, 085406 (2006).

11. Hayashi, D. *et al.* Temperature dependence of the Seebeck coefficient for mixed semiconducting and metallic single-wall carbon nanotube bundles. *Appl. Phys. Express* **13**, 015001 (2020).

12. Nakai, Y. *et al.* Giant Seebeck coefficient in semiconducting single-wall carbon nanotube film Thermoelectric properties of single-wall carbon nanotube films: Effects of diameter and wet environment. *Appl. Phys. Express* **7**, 025103 (2014).

13. Maultzsch, J., Telg, H., Reich, S. & Thomsen, C. Radial breathing mode of single-walled carbon nanotubes: Optical transition energies and chiral-index assignment. *Phys. Rev. B* **72**, 205438 (2005).

14. Zhou, W. Bin *et al.* Ultrahigh-power-factor carbon nanotubes and an ingenious strategy for thermoelectric performance evaluation. *Small* **12**, 3407–3414 (2016).

15. Choi, J. *et al.* Flexible and Robust Thermoelectric Generators Based on All-Carbon Nanotube Yarn without Metal Electrodes. *ACS Nano* **11**, 7608–7614 (2017).

16. An, C. J., Kang, Y. H., Song, H., Jeong, Y. & Cho, S. Y. High-performance flexible thermoelectric generator by control of electronic structure of directly spun carbon nanotube webs with various molecular dopants. *J. Mater. Chem. A* **5**, 15631–15639 (2017).

17. Macleod, B. A., Stanton, N., Gould, I., Wesenberg, D. & Ihly, R. Large n - and p - type thermoelectric power factors from doped semiconducting single - walled carbon nanotube thin films. *Energy Environ. Sci.* **10**, 2168–2179 (2017).

18. Cho, C. *et al.* Completely organic multilayer thin film with thermoelectric power factor rivaling inorganic tellurides. *Adv. Mater.* **27**, 2996–3001 (2015).

19. Cho, C. *et al.* Outstanding Low Temperature Thermoelectric Power Factor from Completely Organic Thin Films Enabled by Multidimensional Conjugated Nanomaterials. *Adv. Energy*

Mater. **6**, 1502168 (2016).

20. Cho, C. *et al.* Organic thermoelectric thin films with large p-type and n-type power factor. *J. Mater. Sci.* **56**, 4291–4304 (2021).

21. Adams, M. J., Verosky, M., Zebarjadi, M. & Heremans, J. P. Active Peltier Coolers Based on Correlated and Magnon-Drag Metals. *Phys. Rev. Appl.* **11**, 054008 (2019).

22. Watzman, S. J. *et al.* Magnon-drag thermopower and Nernst coefficient in Fe, Co, and Ni. *Phys. Rev. B* **94**, 144407 (2016).

23. Rowe, D. M., Kuznetsov, V. L., Kuznetsova, L. A. & Min, G. Electrical and thermal transport properties of intermediate-valence YbAl 3. *J. Phys. D Appl. Phys* **35**, 2183–2186 (2006).

24. Yarbrough, D. W. & Graves, R. S. Transport properties of concentrated Ag-Pd and Cu-Ni alloys from 300-1000 K. in *Thermal Conductivity 16* 319–324 (1983).

25. Boona, S. R. & Morelli, D. T. Enhanced thermoelectric properties of CePd 3-xPt x. *Appl. Phys. Lett.* **101**, 101909 (2012).

26. Sundqvist, B. Thermal diffusivity and thermal conductivity of Chromel, Alumel, and Constantan in the range 100-450 K. *J. Appl. Phys.* **72**, 539–545 (1992).

27. Pollock, D. D. *Thermocouples: theory and properties*. (CRC Press, 1991).

28. Poudel, B. *et al.* High-Thermoelectric Performance of Nanostructured Bismuth Antimony Telluride Bulk Alloys. *Science.* **320**, 634–638 (2008).

29. Ishiwata, S. *et al.* Extremely high electron mobility in a phonon-glass semimetal. *Nat. Mater.* **12**, 512–517 (2013).

30. Pan, Y. *et al.* Mg3(Bi,Sb)2single crystals towards high thermoelectric performance. *Energy Environ. Sci.* **13**, 1717–1724 (2020).

31. Chung, D.-Y. *et al.* CsBi4Te6: A high-performance thermoelectric material for low-temperature applications. *Science.* **287**, 1024–1027 (2000).

32. Behabtu, N. *et al.* Strong, Light, Multifunctional Fibers of Carbon Nanotubes with Ultrahigh Conductivity. *Science.* **339**, 182 (2013).
