# Supplementary material for: Macroscopic weavable fibers of carbon nanotubes with giant thermoelectric power factor
Source: Nat Commun. 2021 Aug 13;12:4931. doi: 10.1038/s41467-021-25208-z (PMC8363648; doi:10.1038/s41467-021-25208-z)
Supplement: Supplementary file 3 — Description of Additional Supplementary Files [file 41467_2021_25208_MOESM3_ESM.pdf]

Description of Additional Supplementary Files

Supplementary Movie 1:

Demonstration of powering an LED using a thermoelectric power generator based on carbon nanotube threads
